# Beta-Cyclodextrin-Decorated Magnetic Activated Carbon as a Sorbent for Extraction and Enrichment of Steroid Hormones (Estrone, β-Estradiol, Hydrocortisone and Progesterone) for Liquid Chromatographic Analysis

**DOI:** 10.3390/molecules27010248

**Published:** 2021-12-31

**Authors:** Anele Mpupa, Azile Nqombolo, Boris Mizaikoff, Philiswa Nosizo Nomngongo

**Affiliations:** 1 Department of Chemical Sciences, Doornfontein Campus, University of Johannesburg, P.O. Box 17011, Johannesburg 2028, South Africa; ampupa@uj.ac.za (A.M.); azilen@uj.ac.za (A.N.); boris.mizaikoff@uni-ulm.de (B.M.); 2 Department of Science and Innovation-National Research Foundation South African Research Chair Initiative (DSI-NRF SARChI), Nanotechnology for Water, University of Johannesburg, Doornfontein 2028, South Africa; 3 Institute of Analytical and Bioanalytical Chemistry, Ulm University, Albert-Einstein-Allee 11, 89081 Ulm, Germany

**Keywords:** magnetic solid phase extraction, progesterone, hydrocortisone, estrone, β-estradiol, wastewater

## Abstract

A β-cyclodextrin-decorated magnetic activated carbon adsorbent was prepared and characterized using various analytical techniques (X-ray diffraction (XRD), scanning electron microscopy–electron diffraction spectroscopy (SEM-EDS) and transmission electron microscopy (TEM)), and the adsorbent was used in the development of a magnetic solid-phase microextraction (MSPE) method for the preconcentration of estrone, β-estradiol, hydrocortisone and progesterone in wastewater and river water samples. This method was optimized using the central composite design in order to determine the experimental parameters affecting the extraction procedure. The quantification of hormones was achieved using high-performance liquid chromatography equipped with a photodiode array detector (HPLC-DAD). Under optimum conditions, the linearity ranged from 0.04 to 300 µg L^−1^ with a correlation of determinations of 0.9969–0.9991. The limits of detection and quantification were between 0.01–0.03 and 0.033–0.1 µg L^−1^, with intraday and interday precisions at 1.1–3.4 and 3.2–4.2. The equilibrium data were best described by the Langmuir isotherm model, and high adsorption capacities (217–294 mg g^−1^) were obtained. The developed procedure demonstrated high potential as an effective technique for use in wastewater samples without significant interferences, and the adsorbent could be reused up to eight times.

## 1. Introduction

Steroids occur naturally in microorganisms, animals and plants. They primarily contain three cyclohexanes, one pentagonal carbon ring attached to different functional groups and side chains [1]. The steroid hormones are all derivatives of cholesterol and are also low-molecular-weight lipophilic compounds [2,3]. Hormones can be broadly classified as either natural or synthetic [3]. Furthermore, they are divided into different families, including mineralocorticoids, glucocorticoids, androgens, estrogens and progesterone [4]. Glucocorticoids have been used as anabolic growth promoters due to their metabolic properties [5]. Hydrocortisone is a corticosteroid belonging to the glucocorticoid family. These types of hormones have found use in human medicine because of their immunosuppressive and anti-inflammatory properties [6,7]. Hydrocortisone is naturally produced by the adrenal cortex as a stress exposure response [8]. Hydrocortisone also takes part in the metabolism of fat, protein and carbohydrates [8]. Estrogens, such as estrone (E1), 17-β-estradiol (E2) and estriol (E3), are steroid hormones that are responsible for the upkeep of the health of breast, skin, brain and reproductive tissues [9]. Physiologically, progesterone (PRO) works as an estrogen hormonal balancer and is involved in the menstrual cycle, pregnancy and embryogenesis [10]. However, high levels of these hormones can increase the risk of osteoporosis, have neurotoxic effects on the central nervous system [5], decrease reproductive performance and the rate of fertilization in fish [11] and have potential carcinogenic effects on humans [3]. They are also considered as an endocrine disruptor, which can cause adverse effects on wildlife and humans by interacting with the endocrine system [12].

In recent years, traces of some steroid hormones have been found in food; these can be traced back to the use of hormones as growth promoters of animals in both industry and farm animals because of their anabolic effects [13,14]. In addition, some steroids (such as progesterone) are used as human and veterinary pharmaceuticals, and as a result, both humans and animals have become sources of environmental pollution. This is of great concern, as the presence of these compounds in food and water negatively affects some organs and systems, such as the cardiovascular system, tissues and the nervous system [15]. Thus, the EU and other countries such as China prohibit the use of hormones because of their potential to be endocrine disruptors in both wildlife and humans [13,15]. While the use of hormones has been banned, they can at times be found in compound mixtures to escape surveillance, thus resulting in residues of such chemicals being found in the environment [16]. As a result, highly sensitive and selective analytical methods are required for the determination of these hormones.

The most common methods for the determination of hormones include chromatographic methods, such as gas chromatography (GC) and liquid chromatography (LC), combined with mass spectrometry detectors for example quadrupole [17]; orbitrap mass spectroscopy [3]; and electrospray ionization mass spectroscopy amongst others [15]. However, due to the complexity of sample matrices, sample preparation is necessary to determine trace hormone residues [16]. Therefore, different sample preparation techniques have been used for the extraction of hormones from various sample matrices. These include, but are not limited to, solid-phase extraction [18], liquid–liquid extraction and hollow fiber liquid-phase microextraction [19]. Solid-phase extraction is one of the commonly used sample preparation techniques [20]. Because of its advantages, such as ease of operation, high enrichment factors and various sorbent materials, SPE is an attractive sample preparation technique for analytical chemists [21].

Activated carbon (AC) is one of the most used materials for the removal and extraction of organic pollutants [22]. Powdered activated carbon can be made from a range of organic materials and is reported to have functional groups that can absorb organic compounds, well-defined porosity and high specific surface areas [23]. Generally, AC is light in weight such that separation from aqueous solutions is a challenge. Thus, for practical application, combining AC powder with magnetic nanoparticles provides simple and quick use and the reuse of AC [24]. Βeta-cyclodextrin (β-CD) is a cyclic oligosaccharide with hydrophobic inner cavities that can form host–guest interactions with the aid of supramolecular interaction between β-CD and the analyte of interest [25,26]. Due to their attractive features, such as unique cavity, cost effectiveness, non-toxicity, biodegradability and renewable properties, β-CD has proven to be an exceptional adsorbent [27,28,29,30]. However, β-CD is highly soluble in water. For this, researchers have resolved this challenge by incorporation into a composite [27,28] or by crosslinking or immobilization [29].

In this study, the combination of magnetic activated carbon and β-CD as an adsorbent for the solid-phase extraction of selected steroid hormones in water samples prior to high-performance liquid chromatography–diode array detector (HPLC-DAD) was reported for the first time. The incorporation of magnetic activated carbon and β-CD solved the problems of water solubility of β-CD and the difficulties of recovering activated carbon after use. In addition, the composite combined the benefits of the hydrophobic cavity of β-CD, the high specific surface area of activated carbon and the magnetic properties of magnetite nanoparticles. The HPLC technique was chosen as an analytical method of choice due to its ease of operation and because no derivation step is required prior to analysis [31]. However, at trace levels, the HPLC technique encounters limitations in the detection of some organic compounds. As a result, a modified yet simple version of SPE was introduced as a sample preparation technique, where SPE was primarily selected for its inclusive nature for the use of modified adsorbents to increase the selectivity of the method [32]. The adsorbent was characterized by X-ray diffraction, scanning electron microscopy and transmission electron microscopy. The parameters affecting the extraction and preconcentration of the steroid hormones were investigated using response surface methodology.

## 2. Materials and Methods

### 2.1. Materials and Reagents

Hydrocortisone (HYD), β-estradiol (E2), progesterone (PRO) and estrone (E1) were obtained from Sigma-Aldrich (St. Louis, MO, USA). The physical properties and chemical structure of the analytes are presented in Table S1. Sodium hydroxide, acetic acid (99.9%), methanol (HPLC grade), ferrous sulphate, ferric (III) chloride and β-cyclodextrin were all purchased from Sigma-Aldrich. A 20 mg L^−1^ mixed hormone stock solution was prepared by dissolving an appropriate amount of the analyte in HPLC-grade methanol and was kept chilled at 2 °C. Standard solutions were prepared daily by diluting the stock solution with ultra-pure water (Direct-Q^®^ 3UV-R purifier system, Millipore, Merck).

### 2.2. Instrumentation

All pH measurements were carried out using an OHAUS ST series pen pH meter. The adsorption studies were carried out using the Branson 5800 Ultrasonic Cleaner (Danbury, CT, USA). A scanning electron microscopy (SEM, TESCAN VEGA 3 XMU, LMH instrument, Tescan Company, Brno, Czech Republic) coupled with energy dispersive X-ray spectroscopy (EDS) was used to study the morphology and elemental composition of the adsorbent at an accelerating voltage of 20 kV. The transmission electron microscopic image was captured using transmission electron microscopy (TEM, JEM-2100, JEOL, Tokyo, Japan).

An Agilent high-performance liquid chromatography (HPLC) 1200 Infinity series, equipped with a photodiode array detector (Agilent Technologies, Waldbronn, Germany), was used for all analyses. The separation was carried out using an Agilent Zorbax Eclipse Plus C18 column (3.5 μm × 150 mm × 4.6 mm) (Agilent, Newport, CA, USA) operated at an oven temperature of 25 °C. The chromatograms were recorded using a 1.00 mL min^−1^ flow rate and solvent mixture of 55% mobile phase A (water) and 45% mobile phase B (acetonitrile), and adsorption wavelengths of 230, 260, 280 and 288 nm using an isocratic elution system were used.

### 2.3. Collection of Samples

Wastewater samples (influent and effluent) were collected from a nearby urban wastewater treatment plant (Gauteng, South Africa) between January 2018 and December 2019. The river water samples were obtained from a river that receives WWTP effluent water. During the study period, 50 influent and effluent samples, as well as 50 downstream river water samples, were collected using the grab sampling technique. All samples were collected using clean 200 mL glass bottles and kept at 2 °C until use. 

### 2.4. Preparation of β-Cyclodextrin-Decorated Magnetic Activated Carbon

Iron solutions of 0.75 mol L^−1^ FeCl_3_ and 0.5 mol L^−1^ FeSO_4_.7H_2_O were prepared separately. The iron solutions were dissolved at a Fe^3+^/Fe^2+^ ratio of 2:1 and stirred for 5 min. Then, 4 g of β-cyclodextrin and activated carbon from waste tires (already prepared by Dimpe and colleagues [33]) were mixed via vigorous stirring and heated at 70 °C. Subsequently, 5 mol L^−1^ NaOH (50 mL) was added to the above solution and heating continued at 70 °C for 30 min. A magnet was used to remove the black precipitate that formed during the changing of the color of the mixture. The resulting magnetic material was washed repetitively with a mixture of ethanol and water (50% v/*v*) solution to eliminate impurities (unreacted materials). Finally, the obtained magnetic material was dried in an oven at 80 °C for 24 h before use.

### 2.5. Ultrasound-Assisted Magnetic Solid-Phase Microextraction Procedure

An appropriate amount of adsorbent (4.3–55.7 mg) was measured accurately into a glass vial with a cap; 5 mL of the sample (with pH adjusted accordingly from 4–9) was then added into the adsorbent. Thereafter, the adsorbent was dispersed using ultrasound in an ultrasonic water bath for the specified amount of time (7–32 min). After the time had elapsed, the supernatant was discarded, with separation achieved with the aid of an external magnet. The analyte was then eluted using an accurate volume (313–1086 µL) of HPLC-grade methanol before analysis.

### 2.6. Optimization of Extraction Procedure

A multivariate optimization approach was used for the optimization of the sample preparation procedure. The optimization strategy was based on a 2^4−1^ fractional factorial design and central composite design to determine the experimental parameters that are significant for the preconcentration of steroid hormones. Table 1 shows the summary of the experimental design conditions for the fractional factorial and central composite designs.

### 2.7. Adsorption and Reusability

The adsorption experiments were carried out according to the method described by Mashile and colleagues [34]. Briefly, 15.9 mg of adsorbent was weighed and transferred into nine sealable glass containers. Then, 5 mL of stock solutions with varying concentrations (namely, 1–10 mg L^−1^) was then added into the adsorbent. Agitation of the solution by an ultrasonic water bath was performed for 20 min; thereafter, the adsorbent and supernatant were separated with the aid of an external magnet. Analysis of the supernatant (1 mL) was then carried out using 1 mL of the HPLC-DAD. In addition, the reusability of the adsorbent was also investigated by appropriate modification of a method described by Nyaba and coworkers [35].

### 2.8. Method Validation

After the determination of the optimum conditions, the developed method was validated according to Hoga and colleagues [36]. Under optimum conditions, the analytical figures of merit of the described method were evaluated using the limits of detection and quantification (LOD and LOQ) using the expressions LOD = (3 × Sd)/s and LOQ = (10 × Sd)/s, where Sd and s are the standard deviation of 10 replicate measurements at the lowest calibration concentration and slope, respectively. The linear ranges (LRs), correlation coefficient (R2) and enrichment factor were also determined using prepared sample solutions in the range of 50–500 µg L^−1^. The precision of the method was also investigated in terms of repeatability and reproducibility using the intraday precision (repeatability) (*n* = 15) and interday precision (reproducibility, *n* = 7 working days). Validation of the developed method was carried out using spiked real sample recoveries due to the absence of certified reference materials (CRM). Briefly, the water samples were spiked at two levels (1 and 5 µg L^−1^). The developed method was then applied to the unspiked and spiked water samples.

## 3. Results and Discussion

### 3.1. Characterization

#### 3.1.1. Scanning Electron Microscopy (SEM) and Energy Dispersive X-ray Spectroscopy (EDS)

**Figure 1:**
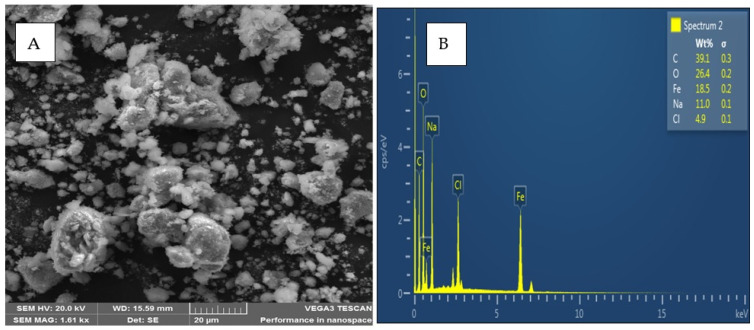
SEM image (**A**) and EDS microgram (**B**) for β-cyclodextrin-decorated magnetic activated carbon.

A morphological analysis of the prepared composite was carried out using SEM. The obtained image is shown in Figure 1A, where small aggregates were observed as a result of the magnetic iron present [37]. Analysis of the material’s elemental composition was performed using EDS, whereby the expected elements resulting from the composite components were observed in their respective peak emissions. The results for the analysis are presented in Figure 1B.

#### 3.1.2. Characterization of Adsorbent by Transmission Electron Microscopy

**Figure 2:**
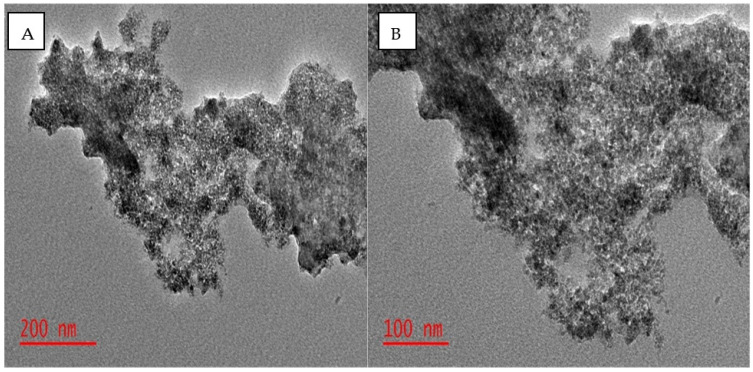
Transmission electron microscopy images of β-cyclodextrin magnetic activated carbon adsorbent at different magnifications: (**A**) 200 nm and (**B**) 100 nm.

Transmission electron microscopy (TEM) was used to study the in-depth structural views of the adsorbent material, and the captured images are presented in Figure 2. The resulting TEM images proved similar to those described by Filippou and coworkers [38], where the iron nanoparticles were attached on the activated carbon powder, which is seen on the image as the darker surface [37]. Similarly, with the SEM images, the material was aggregated as on the TEM capture.

#### 3.1.3. X-ray Diffraction Spectroscopy

**Figure 3:**
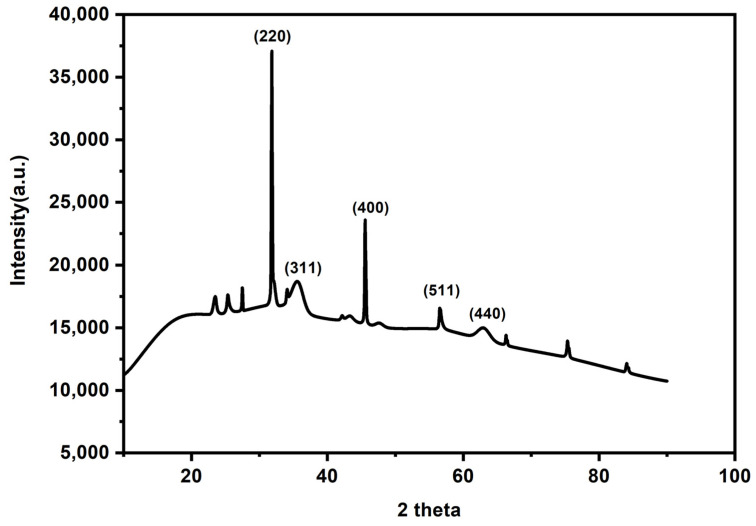
XRD diffraction pattern of the prepared adsorbent material.

To investigate the crystalline structure of the adsorbent, X-ray diffraction was used. Based on the reviewed literature, the expected diffraction peaks are those of the magnetic iron [38] and the activated carbon [33], while β-cyclodextrin did not have an effect on the XRD patterns [39]. The characteristic peaks for the cubic spinel crystal planes of magnetic iron were seen from 2θ = 30.2 (220), 35.56 (311), 43.28 (400, 57.6 (511) and 62.84 (440). The XRD patterns showed (Figure 3) the expected observations as described in the literature.

### 3.2. Optimization of the MSPME Method

#### 3.2.1. Selection of Adsorbent

**Figure 4:**
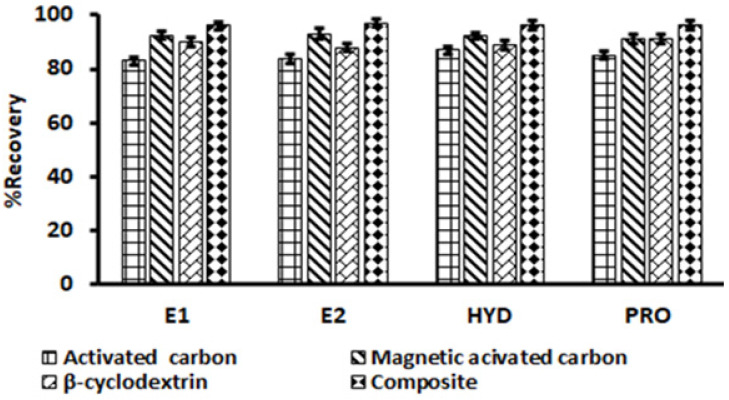
The adsorption performance of activated carbon, magnetic activated carbon, β-cyclodextrin and composite. Experimental conditions: mass of adsorbent (20 mg), extraction time (30 min), eluent volume (1.0 mL), sample pH (6.5), elution time (10 min); sample (5 mL), eluent type (methanol), initial concentration (200 µg L^−1^).

Considering that the activated carbon and cyclodextrin have large surface areas and inherent porous structure, their combination should be able to serve as an ideal adsorbent for the effective extraction of emerging pollutants. In this work, β-cyclodextrin-decorated magnetic activated carbon composite was prepared and used as an adsorbent for the preconcentration of steroid hormones. To investigate the adsorption capabilities of the composite, the performance of the adsorbent for the extraction of selected steroid hormones was compared with the activated carbon, magnetic nanoparticles, β-cyclodextrin and composite under the same conditions. The results shown in Figure 4 revealed that the composite displayed better adsorption capabilities compared to activated carbon, magnetic activated carbon and β-cyclodextrin. The improved adsorption capabilities of the composite for the selected hormones might be due to the combination of the attractive features of each component in the composite.

#### 3.2.2. Screening Process Using Two-Level Fractional Factorial Design

The most influential parameters were screened using a two-level fractional factorial design. In fractional factorial designs, the number of experiments is given by 2^k−p^, where k is the number of variables being studied and p is the indication of fractionation. FFD is used to check the preliminary significance of parameters of interest. This is achieved by estimating the main effects and their interactions while considerably reducing the number of conducted experiments [40]. These factors were the mass of the adsorbent; sample pH; extraction time (ET), which was the time the samples were kept in the ultrasonic bath; and eluent volume (EV). 

**Figure 5:**
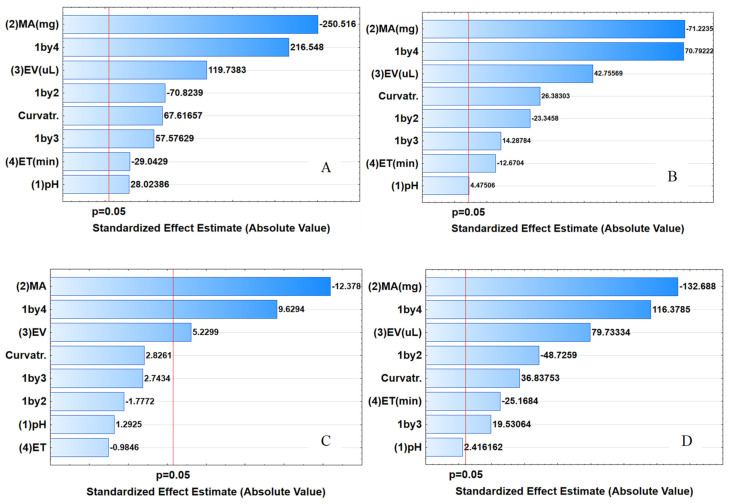
Pareto charts of standardized effects for variables in the preconcentration of steroid hormones (**A**) E1, (**B**) E2, (**C**) HYD and (**D**) PRO. (1) pH of working solution, (2) MA: mass of adsorbent, (3) EV: eluent volume, (4) ET: extraction time, 1by4: pH–ET interaction, 1by2: pH–MA interaction, 1by3: pH–EV interaction.

The design matrix was analyzed with the aid of analysis of variance (ANOVA) to determine which factors and interactions had significance in the extraction of selected steroids. These results are presented in Figure 5 as Pareto charts. A factor is considered significant on a Pareto chart when the bar of that respective factor crosses the red line, which is considered as the 95% confidence level [41,42]. In this case, according to the obtained Pareto chart, the factors that affected the extraction of E1, E2 and PRO were MA, ET and EV and the interaction between pH and EV. The preconcentration of HYD, MA and EV and the interaction between pH and EV were significant at the 95% confidence level. The effect of pH was found to be significant for E1 but not significant for E2, HYD and PRO. This could be a result of the formation of an inclusion complex formed by β-cyclodextrin’s interior cavity and the analyte in its molecular state (pH lower than pK_a_, pK_a_ presented in Table S1) resulting in hydrogen bond interactions as well as π–π interactions between the analyte and glucose monomers of β-cyclodextrin [43,44]. Thus, with further optimization, the central composite design (CCD) was used to optimize the effect of MA, ET and EV.

#### 3.2.3. Response Surface Methodology and Desirability Function

**Figure 6:**
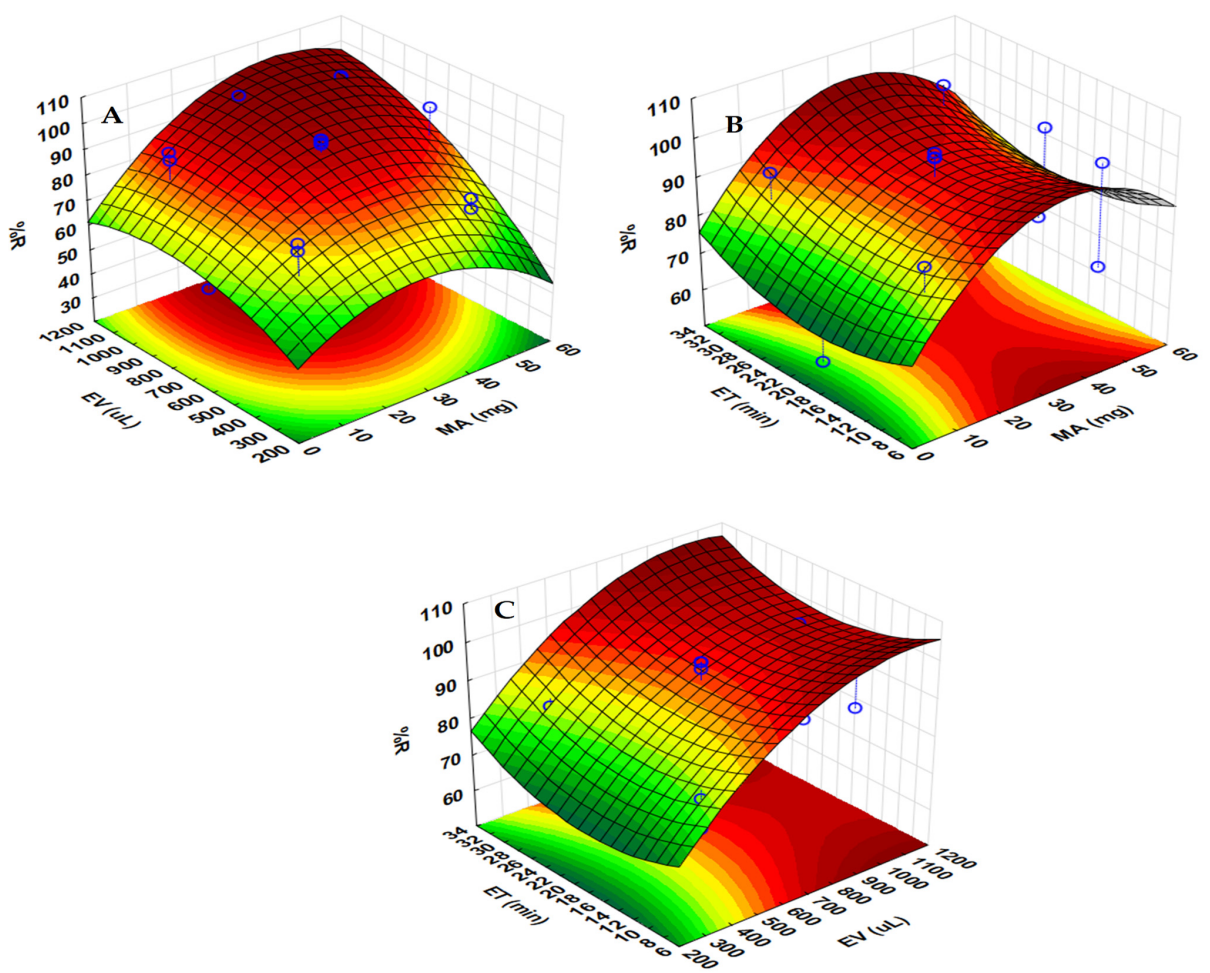
Three-dimensional response surface plot showing the result (E1) of the interaction of (**A**) eluent volume (EV) and mass of adsorbent (MA), (**B**) extraction time (ET) and MA and (**C**) EV and ET on % R.

For further optimization of the significant factors, the central composite design method based on the surface response methodology (RSM) was used, where the three parameters (namely, MA, ET and EV) were optimized. Figure 6A,B show the combined effect of MA with ET and EV on the % R. The results obtained indicate that better recoveries were obtained when the mass of adsorbent was greater than or equal to 10 mg. In addition, Figure 6A, C demonstrated that quantitative recoveries of target analytes were possible with an EV ranging from 400 to 1000 µL. The extraction time was found to be insignificant, suggesting that any time above zero could lead to quantitative recoveries. Finally, the interactions between ET and EV (Figure 6C), as well as ET and MA, show that EV and MA (Figure 6B) were the main contributors in the extraction and preconcentration of steroid hormones. The RSM plots for E2, HYD and PRO are presented in Figure S1.

**Figure 7:**
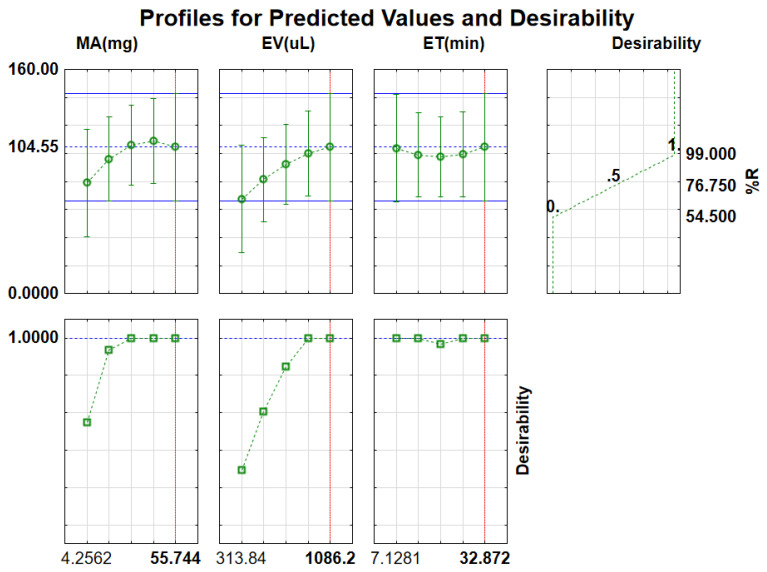
Profiles for desirability for the extraction of E1.

The 3D response surface plots shown in Figure 6, in addition to the profiles for desirability (Figure 7), were also used to determine the optimum conditions for the extraction procedure for E1 (desirability plots for E2, HYD and PRO are presented in Figure S2). Herein, a desirability score of 1.00 was assigned to a maximum recovery of 98.1%, 77.1% was assigned to a desirability of 0.5, and a minimum % R of 55.4% was associated with a desirability of 0. A score of 1 for the factors was associated with the value required to achieve maximum extraction recovery [45]. The values for each factor were found to be 30 mg for MA, 893 µL for EV and 32 min for ET. Therefore, the overall optimum conditions used for the extraction procedure were a pH, MA, ET and EV of 7, 30 mg, 32 min and 893 µL, respectively. The determined optimum conditions were validated experimentally, and the determined recovery was 99.3 ± 0.4, which agrees with the RSM model prediction at the 95% confidence level.

#### 3.2.4. Analytical Performance of MSPME-HPLC-DAD Method

Under optimum conditions, the analytical figures of merit of the described method were evaluated using the limits of detection and quantification (LOD and LOQ). The linear ranges (LRs), correlation coefficient (R^2^) and preconcentration factor were also determined using prepared sample solutions and are presented in Table 2. The method was found to be linear for the analytes in the range LOQ—300 µg L^−1^ with correlation coefficients (R^2^) 0.9988, 0.9991, 0.9969 and 0.9987 for E1, E2, HYD and PRO, respectively. The LODs for E1, E2, HYD and PRO were found to be 0.01, 0.02, 0.03 and 0.02 µg L^−1^, while the LOQs for the same compounds were 0.033, 0.067, 0.1 and 0.067 µg L^−1^, respectively. The method precision in terms of the interday and intraday was found to be 2.3, 2.5, 1.1 and 3.4 and 4.4, 3.5, 3.2 and 4.2 for E1, E2, HYD and PRO, respectively. The enrichment factors for the analytes were 93, 81, 90 and 91. The performance of the method was then further compared to other solid-phase methods reported in the literature in Table 3. The current method was found to have better LOD and precision compared to those reported by Liao and colleagues and Sampaio and coworkers [46,47]. Furthermore, the analytical performance was comparable to that reported by other researchers [48,49,50]. However, the LOD was higher than that reported elsewhere [6,51].

#### 3.2.5. Validation and Application

##### Spike Recovery Test

In order to validate the proposed method, spiked influent wastewater samples and spiked recoveries were preferred due to the unavailability of certified reference material (CRM) for hormones. The results that are presented in Table 4 show that the proposed method showed good recoveries of 95–99.1% in influent, 93.5–102% in effluent and 96.6–103% in river water.

##### Determination of Selected Steroid Hormones in Wastewater (Influent and Effluent) and River Samples

Based on the acceptable recovery test results, the developed analytical method was applied for the extraction and preconcentration of steroid hormones in influent, effluent and river water samples collected from an urban wastewater treatment and nearby river. The obtained results are summarized in Table 5. The results showed that E1, E2 and PRO were detected in wastewater influent and effluent, as well as river water samples, while HYD was only detected in wastewater samples. In the wastewater samples, the mean concentrations ranged from 32.3 to 35.9 ng L^−1^, 1132 to 2414 ng L^−1^, 1.07 to 2.50 ng L^−1^ and 3.92 to 6.35 ng L^−1^ for E1, E2, HYD and PRO, respectively. The presence of E1, E2 and PRO in the river water samples could be due to the incomplete removal of these hormones during the treatment process. The obtained results proved that combining a less sensitive chromatographic technique such as HPLC-DAD with MSPME (preconcentration method) could lead to an accurate quantification of E1, E2, HYD and PRO at the ultratrace level (nanograms per liter levels). A typical chromatogram for the determination of target analytes in real water samples is shown in Figure S3.

The results obtained in this study were compared with those reported by other researchers. Mhuka and colleagues [53] reported E1, E2, PRO concentrations in South African influent (E1: < LOD-35.96 ng L^−1^; E2: 66.45–2206 ng L^−1^; PRO: LOD-14.5 ng L^−1^), effluent (E1: < LOD-60.83 ng L^−1^; E2: 154.1–7133 ng L^−1^; PRO: LOD-4.03 ng L^−1^) and river water (E1: 7.12–63.04 ng L^−1^; E2: 134.7–644 ng L^−1^; PRO: LOD-2.20 ng L^−1^). These values are comparable or higher than the results obtained in the current study. Merlo and colleagues [6] conducted a study on the determination of E1, E2 and PRO in river water and wastewater treatment plant (WWTP) effluent collected from high-density populated areas of northern Italy. Their concentrations (E1: 14–76 ng L^−1^; E2 < LOQ-34 ng L^−1^; PRO: 4–10 ng L^−1^) were lower than the ones obtained in this study. The concentrations of E1 and E2 in Chilean wastewater (influent and effluent) samples were found to be 15–16 and 0–48 ng L^−1^ [51]. Sampaio and colleagues [47] investigated E1, E2 and PRO at concentrations ranging from 1110 to 1580 ng L^−1^ in Brazilian surface water samples.

#### 3.2.6. Adsorption and Reusability

##### Adsorption Studies

Equilibrium adsorption data for the proposed method were investigated using Langmuir [54] and Freundlich [55] models; linearized equations for each model were used and are shown in Equations (1) and (2), respectively:

(1) $$\frac{C_{e}}{q_{e}}=\frac{1}{q_{max}}C_{e}+\frac{1}{K_{L}q_{max}}$$

where *q_e_* is the equilibrium adsorption capacity (mg g^−1^), *q_max_* is the maximum monolayer adsorption capacity (mg g^−1^), *K_L_* is the Langmuir constant (L mg^−1^), and *C_e_* is the concentration of adsorbate at equilibrium (mg L^−1^).

(2) $$lnq_{e}=lnK_{F}+\frac{1}{n}lnC_{e}$$

where *K_F_* is the measure of adsorption capacity, *C_e_* is the concentration of adsorbate at equilibrium (mg L^−1^), and *n* is an adsorption effectiveness indicator.

**Figure 8:**
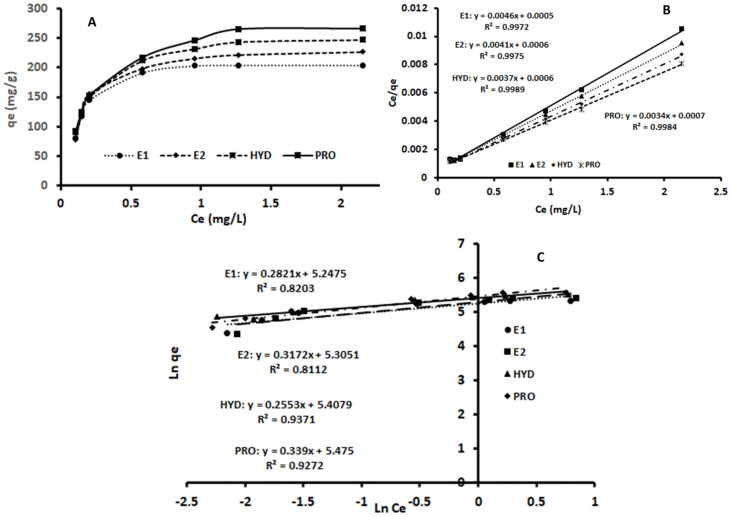
Plots of *C_e_/q_e_* against *C_e_* (**A**), and *ln q_e_* versus *ln C_e_*, for Langmuir (**B**) and Freundlich (**C**) models.

The plots of *C_e_/q_e_* against *C_e_*, and *ln q_e_* versus *ln C_e_*, for the Langmuir and Freundlich models are presented in Figure 8. The correlation coefficients (R^2^) are presented in Table 6 and show that the R^2^ values for the Langmuir models are higher than the Freundlich models. This implies that the adsorption of the steroid hormones took place at particular homogeneous sites and as a monolayer adsorption onto the surface of the magnetic adsorbent [56]. The values of *q_max_* and *K_L_* were obtained from the intercept and slope of the plot (Figure 8), and they were found to be 217 mg g^-1^ and 9.2 L mg^−1^, 244 mg g^−1^ and 6.8 L mg^−1^, 270 mg g^−1^ and 6.2 L mg^−1^ and 294 mg g^−1^ and 4.9 L mg^−1^ for E1, E2, HYD and PRO, respectively.

##### Reusability and Regeneration

**Figure 9:**
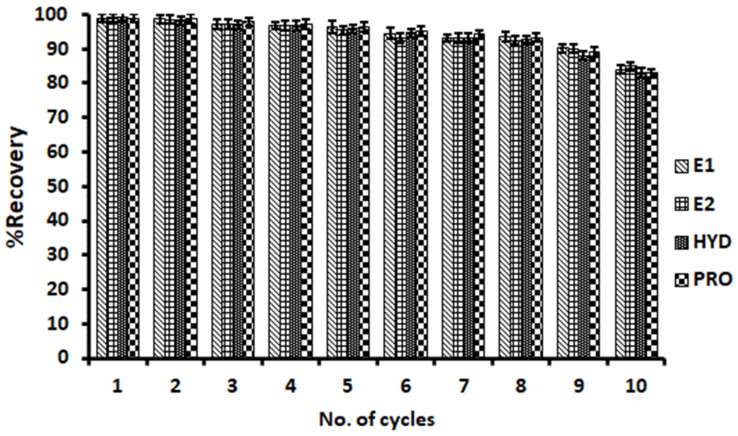
Regeneration of β-cyclodextrin-decorated magnetic activated carbon composite (initial concentration: C_0_ = 100 µg L^−1^) (*n* = 3).

The reusability and regeneration of the β-cyclodextrin-decorated magnetic activated carbon composite were studied in order to investigate its practical application in water treatment processes. The regenerated and recycled results of the magnetic adsorbent (Figure 9) demonstrated that the adsorption/desorption (recovery) cycles for E1, E2, HYD and PRO solutions were not affected for up to eight runs of the regenerated magnetic adsorbent. This phenomenon suggested that the as-prepared adsorbent could be easily recovered and reused effectively. Therefore, β-cyclodextrin-decorated magnetic activated carbon composite has the potential for application in the determination of steroid hormones in water.

## 4. Conclusions

Even though the use of some steroid hormones was banned due to their endocrine disruptor abilities, traces of these pollutants are still found in different water matrices. As such, analytical methods able to determine these compounds from complex matrices are of urgent necessity. In this work, a simple and effective magnetic solid-phase microextraction procedure was developed, which showed comparable analytical performance to other solid-phase extraction-based methods found in the previous literature. The adsorption of steroid hormones onto the adsorbent was found to be described by the Langmuir adsorption isotherm, with high maximum adsorption capacities of 217, 244, 270 and 294 mg g^−1^ for E1, E2, HYD and PRO. Moreover, the adsorbent could be used up to eight times while maintaining a 92% recovery.

## Figures and Tables

**Table 1:** Parameter and levels used in 2^4−1^ fractional factorial design and central composite design.

Factors	−α	Low Level (−1)	Central Point (0)	High Level (+1)	+α
Mass of adsorbent (MA) (mg)		10	30	50	
pH		4	6.5	9	
Extraction time (ET) (min)		10	20	30	
Eluent volume (µL)		400	700	1000	
Central composite design	**−α**				**+α**
Mass of adsorbent (MA) (mg)	4.25	10	30	50	55.74
Extraction time (ET) (min)	7.12	10	20	30	32.87
Eluent volume (µL)	313.8	400	700	1000	1086

**Table 2:** Analytical performance of the developed preconcentration method.

Analytical Characteristics	E1	E2	HYD	PRO
Linearity (µg L^−1^)	0.04–250	0.7–300	0.1–250	0.07–200
Correlation coefficient (R^2^)	0.9988	0.9991	0.9969	0.9987
Limits of detection (LOD) (µg L^−1^)	0.01	0.02	0.03	0.02
Limits of quantification (LOQ) (µg L^−1^)	0.033	0.067	0.10	0.067
Enrichment factor	93 ± 2	81 ± 3	90 ± 2	91 ± 2
Recovery (%, mean ± SD, *n* = 6)	99.1 ± 2.5	98.5 ± 3.1	98.9 ± 2.1	97.5 ± 3.1
Intraday precision (*n* = 10 measurements), %	2.3	2.5	1.1	3.4
Interday precision (*n* = 5 working days), %	4.4	3.5	3.2	4.2

**Table 3:** Analytical performance comparison between current method and literature-reported methods.

Analyte	Matrix	Analytical Technique	LOD (µg L^−1^)	RSD	Reference
Steroid hormones	Water samples	SPE-LC-APCI-MS/MS	0.0058–0.015	1–22	[48]
E2	Water and urine samples	SPME-HPLC	0.12	<8	[46]
E1, E2	wastewater	SPE-GC-MS	0.011–0.060	0.10–0.28	[49]
Estradiol, testosterone, PRO, HYD	River water	ETA-SHS-ME-HPLC-UV	0.002–0.0017		[52]
E1, E2	Wastewater	SPE-HPLC-MS	0.004–0.014		[50]
E1, E2	Wastewater	RDSE-GC-MS	0.003–0.006	5–9	[51]
E1, E2, PRO	Surface water	SPE-GC-MS	0.13–0.3	0.2–22	[47]
E1, E2, PRO	Wastewater and river water	SPE-HPLC-MS/MS	0.00002–0.0009	<15	[6]
E1, E2, HYD, PRO	Wastewater and river water	MSPME-HPLC-DAD	0.01–0.03	2.1–3.1	Current work

**Table 4:** Validation parameters of the preconcentration method in water samples (*n* = 3).

Analytes		Influent		Effluent		River	
	Added (µg L^−1^)	Found (µg L^−1^)	% R	Found (µg L^−1^)	% R	Found (µg L^−1^)	% R
E1	0	0.120 ± 0.005		0.0631 ± 0.001		0.037 ± 0.001	
	1	1.07 ± 0.04	95.2 ± 3.7	1.03 ± 0.02	97.1 ± 1.9	1.02 ± 0.03	98.2 ± 2.9
	5	4.96 ± 0.12	96.7 ± 2.4	4.98 ± 0.15	98.3 ± 1.2	4.96 ± 0.13	98.5 ± 2.6
E2	0	2.41 ± 0.11		1.67 ± 0.02		0.52 ± 0.01	
	1	3.39 ± 0.08	97.8 ± 2.4	2.65 ± 0.05	98.3 ± 1.9	1.51 ± 0.05	98.5 ± 3.3
	5	7.37 ± 0.14	99.1 ± 1.9	6.77 ± 0.21	102 ± 3.1	5.67 ± 0.21	103 ± 3.7
HYD	0	ND		ND		ND	
	1	0.96 ± 0.03	95.6 ± 3.1	0.97 ± 0.04	96.7 ± 4.1	0.99 ± 0.04	98.5 ± 4.1
	5	4.87 ± 0.11	97.3 ± 2.6	4.92 ± 0.13	98.4 ± 2.6	4.97 ± 0.13	99.3 ± 2.6
PRO	0	ND		ND		ND	
	1	0.92 ± 0.01	92.3 ± 1.1	0.94 ± 0.02	93.5 ± 2.1	0.97 ± 0.04	96.6 ± 4.1
	5	4.78 ± 0.14	95.5 ± 2.9	4.82 ± 0.17	96.3 ± 3.5	4.88 ± 0.17	97.6 ± 3.5

**Table 5:** Minimum (min), maximum (max) and mean concentration (ng L^−1^) of four steroid hormones in influent wastewater, effluent wastewater and river water samples: *n*= number of samples containing detectable amounts of target analytes.

Analytes	Influent Wastewater (*n* = 35)			Effluent Wastewater (*n* = 35)			River Water (*n* = 20)		
	Min	Max	Mean	Min	Max	Mean	Min	Max	Mean
E1	15.7	126	35.9	10.4	57.8	32.3	10.4	63.1	30.0
E2	143	6234	2414	67.4	2207	1132	124	948	463
HYD	<LOQ	87.5	2.50	<LOQ	37.3	1.07	<LOQ	<LOQ	<LOQ
PRO	<LOQ	127	6.35	<LOQ	78.3	3.92	<LOQ	68.3	3.42

**Table 6:** Langmuir and Freundlich adsorption models and parameters investigated in the study.

	Parameters	E1	E2	HYD	PRO
Langmuir	*q_max_* (mg g^−1^)	217	244	270	294
	*K_L_* (L mg^−1^)	9.2	6.8	6.2	4.9
	R^2^	0.9972	0.9975	0.9989	0.9984
Freundlich	*K_F_*	190	201	223	239
	*n*	3.5	3.2	3.9	2.9
	R^2^	0.8203	0.8112	0.9371	0.9271

## Supplementary Materials

The following are available online, Table S1: Structural and physical properties of target analytes, Figure S1: Three-dimensional response surface plot showing the result (E2 (A-C), HYD (D-F) and PRO (G-I)) of the interaction of (A) eluent volume (EV) and mass of adsorbent (MA), (B) extraction time (ET) and MA and (C) EV and ET on % R, Figure S2: Profiles for desirability for the extraction of E2, HYD and PRO, Figure S3: Typical chromatogram of steroid hormones showing peaks for 1: estrone, 2: β-estradiol, 3: hydrocortisone and 4: progesterone at 250 nm. Flow rate of 1.00 mL min^-1^ and solvent mixture of 55% mobile phase A (water) and 45% mobile phase B (acetonitrile), using an isocratic elution system.

## Sample Availability

Samples of the compounds are available from the authors.
