# Supplementary material for: Beta-Cyclodextrin-Decorated Magnetic Activated Carbon as a Sorbent for Extraction and Enrichment of Steroid Hormones (Estrone, β-Estradiol, Hydrocortisone and Progesterone) for Liquid Chromatographic Analysis
Source: Molecules. 2021 Dec 31;27(1):248. doi: 10.3390/molecules27010248 (PMC8747044; doi:10.3390/molecules27010248)
Supplement: Supplementary file 1 [file molecules-27-00248-s001.zip › molecules-1501981-supplementary.pdf]

Beta-Cyclodextrin-Decorated Magnetic Activated Carbon as a Sorbent for Extraction and Enrichment of Steroid Hormones (Estrone, β-Estradiol, Hydrocortisone and Progesterone) for Liquid Chromatographic Analysis

Anele Mpupa [1,2], Azile Nqombolo [1,2], Boris Mizaikoff [1,3] and Philiswa Nosizo Nomngongo [1,2,*]

[1] Department of Chemical Sciences, Doornfontein Campus, University of Johannesburg, P.O. Box 17011, Johannesburg 2028, South Africa; ampupa@uj.ac.za (A.M.); azilen@uj.ac.za (A.N.); boris.mizaikoff@uni-ulm.de (B.M.);

[2] Department of Science and Innovation-National Research Foundation South African Research Chair Initiative (DSI-NRF SARChI): Nanotechnology for Water, University of Johannesburg, Doornfontein 2028, South Africa

[3] Institute of Analytical and Bioanalytical Chemistry, Ulm University, Albert-Einstein-Allee 11, 89081 Ulm, Germany

* Correspondence: pnnomngongo@uj.ac.za; Tel.: +27-11-559-6571

Table S1. Structural and physical properties of target analytes.

Compound	Molecular weight (g/mol)	pK_a	Log K_{ow}
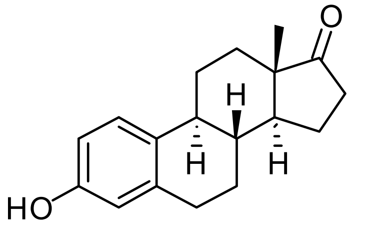 **Estrone (E1)**	270.37	10.7	4.1
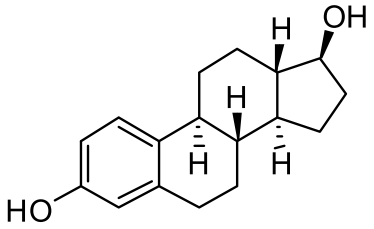 **Estradiol (E2)**	272.38	10.7	3.7

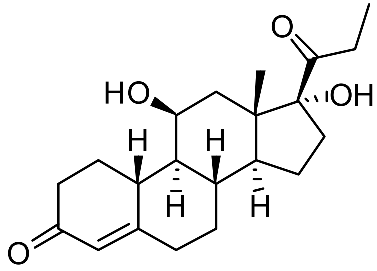 **Hydrocortisone (HYD)**	362.46	13.8	1.6
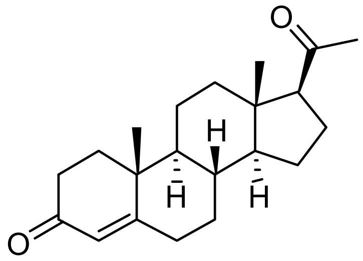 **Progesterone (PRO)**	270.37	18.9	3.9

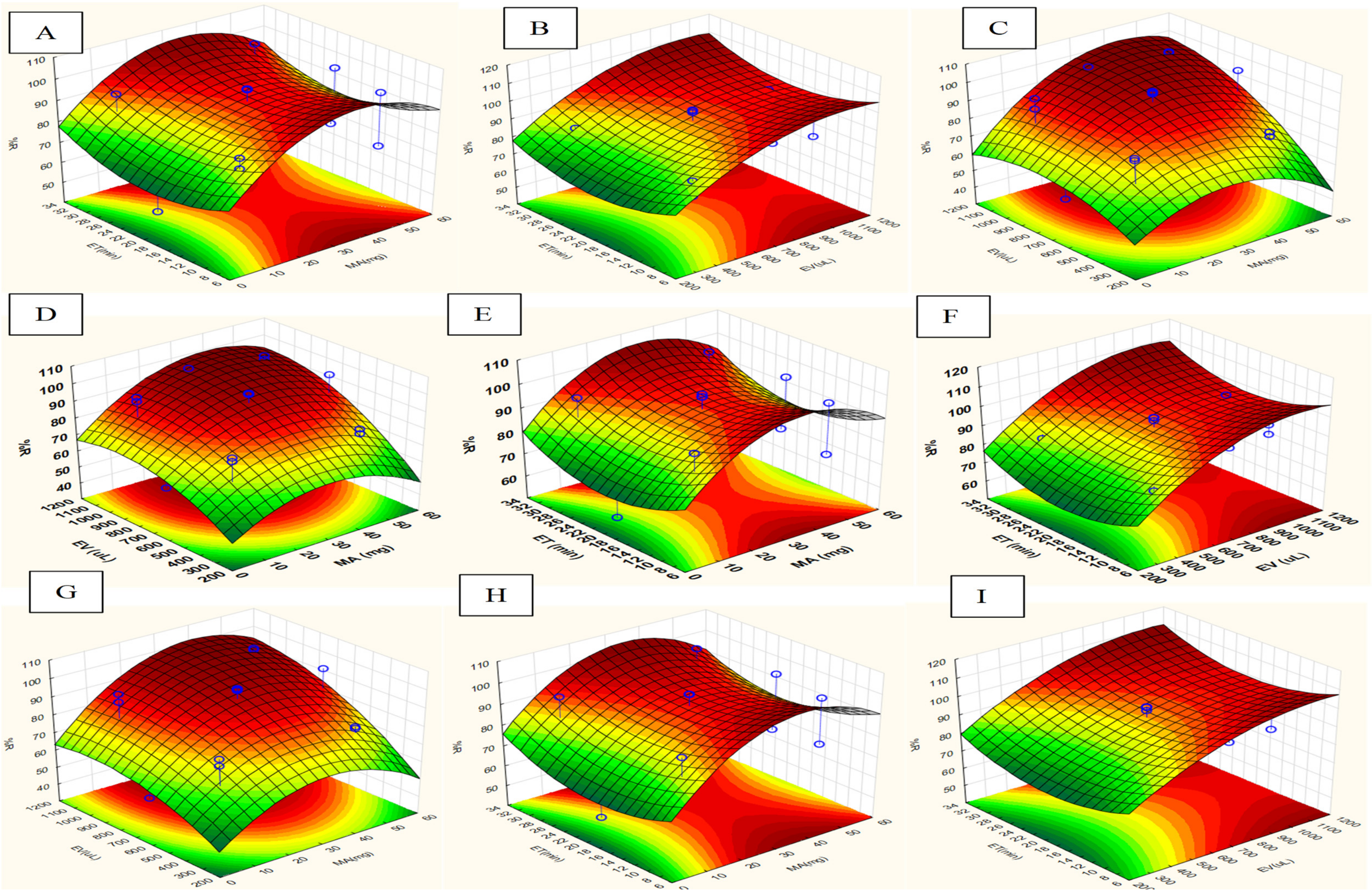

Figure S1. Three dimensional response surface plot showing the result (E2 (A-C), HYD (D-F) and PRO (G-I) of the interaction of (A) eluent volume (EV) and mass of adsorbent (MA), (B) extraction time (ET) and MA and (C) EV and ET on % R.

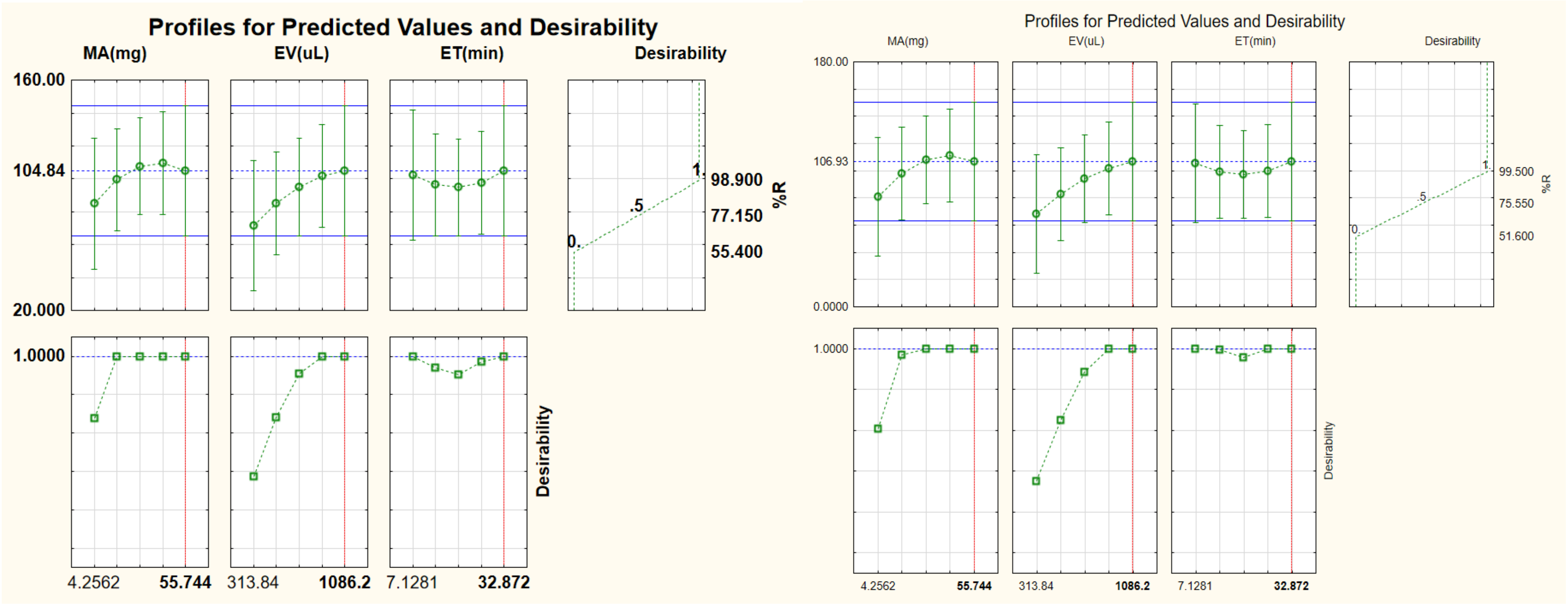

Profiles for Predicted Values and Desirability
MA(mg)
EV(uL)
ET(min)
Desirability
160.00
104.84
20.000
1.0000
98.900
77.150
55.400
%R
Desirability
4.2562
55.744
313.84
1086.2
7.1281
32.872
Profiles for Predicted Values and Desirability
180.00
106.93
0.0000
99.500
75.550
51.600

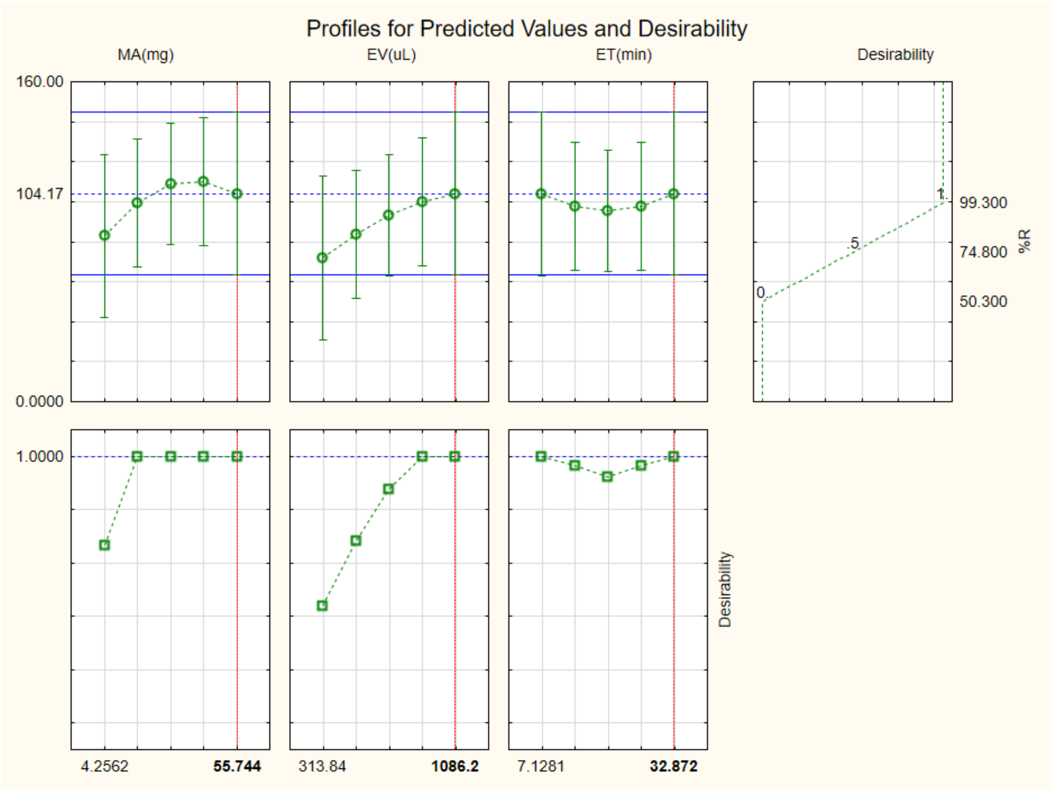

Figure S2. Profiles for desirability for the extraction of E2, HYD and PRO.

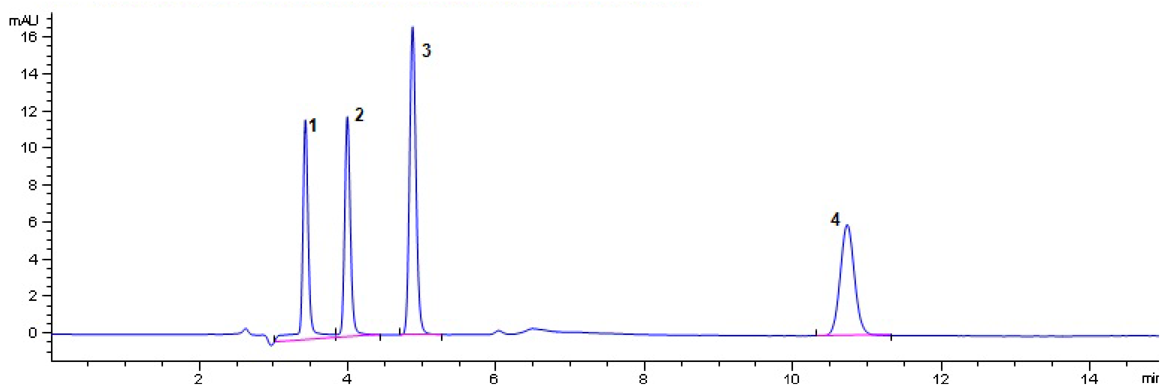

Figure S3. Typical chromatogram of steroid hormones showing peaks for 1: estrone, 2: β-estradiol, 3: hydrocortisone and 4: progesterone at 250 nm. 1.00 mL min-1 flow rate, solvent mixture of 55 % mobile phase A (water) and 45 % mobile phase B (acetonitrile), using an isocratic elution system.
